# Event and Comment

**Published:** 1915-06

**Authors:** 


### DENTAL REGISTER

Vol. LXIX JUNE 1915 No. 6

### EVENT AND COMMENT.

SHALL THE ADDED YEAR TO THE DENTAL CURRICULUM BE A REAL ADVANCE? Already much speculation has been had as to the content of the new college course, and there seems to be as much variety of opinion as there have been suggestions. The discussion, however, seems to be lining up into two camps. On one side there is an inclination to urge an increase in the time devoted to instruction in technical and clinical dentistry, with little increase in time devoted to the fundamental subjects. On the other side there is a strong opinion, especially among college instructors that more time should be devoted to instruction in the fundamental sciences and the purer cultural subjects. We can readily understand the attitude of the dentist who has not been long, or not at all, a teacher, as his mind is running all the time in the rut of the technical, and he has never studied comparatively the results of pedagogic discipline in cultivating a mental acumen that would give one a substantial basis for initiative in technical development. As a rule the better the training a man has in fundamental science, the better thinker he will make and the more reliable will be his conclusions even when directed along the line of technics. A dental student who is allowed to spend the major part of his college course in the development of desirable technique at the expense of thorough grounding in the sciences on which his technique is based, will not make as successful a practioner as he should be with his education more evenly balanced. In dental colleges, as at present conducted, the idea seems to prevail that a student must be taught all the technical operations the time allows, consequently we have developed good artisans but poor thinkers.

A student who has passed through a curriculum which is logically arranged to produce men of understanding, as well as men of reasonable technical skill, will not stop growing when he leaves college. He will have learned to do his own thinking, and he will read and listen to wiser men than he is, and will become a better professional man, because he is continually developing both sides of his nature. Colleges should not be expected to turn out a finished article in the graduated student, but it should be their aim to start their student on the right method of a full self-development.

We have for so long a time emphasized the technical in our training that we have established convictions that our profession would not make satisfactory progress unless this phase of training the dentist receives special encouragement. Is it not possible that the time has come for a new adjustment of our ideals and methods? Just how this is to be done we can only suggest. Some of the stronger schools seem inclined to think, judging by the schedules suggested by them, that more emphasis should be placed on the study of the fundamental sciences like general biology, physics, and chemistry, believing that time so spent will give dental students a broader culture, and better prepare them to assimilate their strictly dental studies and so amply justify the time spent on them. In this connection we should not forget that the best part of a dentist's education is had after he enters upon the practice of his profession; consequently it should be more important that he be intelligent than that he be skillful when he graduates.

A SILVER LOVING CUP FOR THE DONORS OF THE FORSYTH INFIRMARY.—We cordially commend the following suggestion by Dr. Belcher, Editor of *Oral Hygiene,* and trust the profession may make a liberal response.

In the building and endowment of the Forsyth Dental Infirmary for Children, the dental profession has received an uplift that is world wide. No one who views this institution, erected for the betterment of uncounted generations of children yet unborn, can but be impressed with the unselfish character of the man who made the gift and also the two brothers left to carry out his wishes. One of these has since passed away and there remains Thomas A. Forsyth, whose duty and pleasure it has been to add to and embellish the original plan. Today this institution stands as a permanent memorial with an endowment of a million and a half, enabling the trustees to not only conduct the work of caring for the teeth of the worthy poor but to enter the research field and thus it is to be a beacon light and standard so long as it shall endure. The editor suggests that the dental profession secure by subscription among its members, an amount of money sufficient to purchase a beautiful loving cup which shall be placed in the donor's room of the Forsyth Dental Infirmary and there remain for all time, a token of our appreciation. To properly represent the dental profession it should have the united support and indorsement of every dental society and dental journal in the land.

Will you present this subject before the next meeting of your state society and see that it is brought before the meeting of the National Dental Association and the Panama-Pacific Dental Congress? Do not let us adjourn these meetings without taking steps that will make the presentation of a loving cup an accomplished fact. To do less will be a disgrace.

